# Flow cytometry for near-patient testing in premature neonates reveals variation in platelet function: a novel approach to guide platelet transfusion

**DOI:** 10.1038/s41390-019-0316-9

**Published:** 2019-01-29

**Authors:** Amie K. Waller, Lajos Lantos, Audrienne Sammut, Burak Salgin, Harriet McKinney, Holly R. Foster, Neline Kriek, Jonathan M. Gibbins, Simon J. Stanworth, Stephen F. Garner, Vidheya Venkatesh, Anna Curley, Gusztav Belteki, Cedric Ghevaert

**Affiliations:** 1 Department of Haematology, University of Cambridge, Cambridge, Cambridgeshire UK; 2 The Rosie Hospital, Cambridge University Hospitals, Cambridge, Cambridgeshire UK; 3 The Institute for Cardiovascular and Metabolic Research, University of Reading, Reading, UK; 4 Transfusion Medicine, NHS Blood and Transplant, Oxford, UK; 5 NHS Blood and Transplant, Cambridge, Cambridgeshire UK

## Abstract

**Background:**

Neonatal haemorrhaging is often co-observed with thrombocytopenia; however, no evidence of a causal relationship with low platelet count has been reported. Regardless, the administration of a platelet transfusion is often based upon this parameter. Accurate measurement of platelet function in small volumes of adult blood samples by flow cytometry is well established and we propose that the use of the same technology could provide complementary information to guide the administration of platelet transfusions in premature neonates.

**Methods:**

In 28 neonates born at 27–41 weeks gestation, platelet function after stimulation agonists was measured using fibrinogen binding and P-selectin expression (a marker of degranulation).

**Results:**

Platelets of neonates with gestation of ≤36 weeks (*n* = 20) showed reduced fibrinogen binding and degranulation with ADP, and reduced degranulation with CRP-XL. Degranulation Scores of 7837 ± 5548, 22,408 ± 5301 and 53,131 ± 12,102 (mean ± SEM) identified significant differences between three groups: <29, 29–36 and >36 weeks gestation). Fibrinogen binding and degranulation responses to ADP were significantly reduced in suspected septic neonates (*n* = 6) and the Fibrinogen Binding scores clearly separated the septic and healthy group (88.2 ± 10.3 vs 38.6 ± 12.2, *P* = 0.03).

**Conclusions:**

Flow cytometric measurement of platelet function identified clinically different neonatal groups and may eventually contribute to assessment of neonates requiring platelet transfusion.

## Introduction

Neonatal thrombocytopenia and bleeding are common clinical problems for preterm neonates, such that around a quarter of neonates admitted to Neonatal Intensive Care Units (NICUs) develop thrombocytopenia. But the close temporal association commonly noted in sick babies between low platelet counts and the occurrence of clinical bleeding does not in itself establish cause and effect, and one study reported no evidence of a relationship between platelet count and occurrence of major haemorrhage.^1^ Ninety-one percent of neonates with platelet counts below 20 × 10^9^/L did not develop major haemorrhage. In a follow-up analysis of all bleeding outcomes in the previous study, gestational age <34 weeks, development of severe thrombocytopenia within 10 days of birth and necrotizing enterocolitis were found to be the strongest predictors for an increased number of minor bleeding events. A lower platelet count, in itself, was not a strong predictor of increased bleeding risk.^2^ In earlier studies investigating the effect of neonatal thrombocytopenia on bleeding times, only a moderate correlation was observed, but the validity of these findings is limited by poor reproducibility and operator dependency inherent to the methods used to assess functional haemostasis in neonates.^3^ Similarly, in adult patients with haematological malignancies, the platelet count alone appears to be a poor predictor of bleeding risk.^4^

Despite the evidence that the platelet count by itself has limitations as an effective marker of the need for platelet transfusion, it is the most commonly applied parameter used in premature infants to guide platelet administration. There is a need to identify better laboratory tests of bleeding risk, other than provided by platelet count alone, which fails to provide information on platelet function.

Most current tests of platelet function require a volume of blood that is not feasible to collect from premature infants (Table 1). One possible technique is to assess platelet function by flow cytometry, which requires much smaller volumes of blood. The use of flow cytometry for the measurement of platelet function has been used in a series of studies in adults,^5–7^ and has been shown to be sensitive and reproducible enough to clearly identify individuals who have more or less reactive platelets amongst a large cohort of healthy individuals.^8,9^ Due to the size of the premature neonates who are most at risk of bleeding, the use of flow cytometry to measure platelet function is attractive as the assay can be carried out using extremely small samples of whole blood (e.g. <500 µL). Several studies of platelet function by flow cytometry have been carried out in the neonatal population suggesting hyporeactivity of neonatal platelets compared to those of adults, potentially related to the lower expression of key platelet surface receptors.^10^ This hyporeactivity has been reported to peak in the first few days post delivery before gradually correcting over the first few weeks of life.^11^ Most of these studies have been carried out in healthy newborns using cord blood samples, although there are still doubts that platelets function from cord blood or peripheral blood are truly comparable.^12^ Even fewer studies have analysed platelet function in preterm newborns, although the data so far suggest that very low birth weight/premature babies have impaired platelet function that improves with gestational age.^13–16^ It is thought that this correlates with the “developmental progression” from embryonic to foetal and finally adult haematopoiesis imposing inherent differences to the megakaryocytes and their platelet progeny.^17,18^

**Table 1:** Clinical and non-clinical assessment of platelet function

Clinical			Non-clinical		
Name of test	Method	Blood requirement	Name of test	Method	Blood requirement
Light transmission aggregometry	Platelet Rich Plasma is stirred in a cuvette at 37 °C. When an agonist is added, the platelets aggregate increasing light transmission which is detected by a photocell.	>5 mL	Light transmission aggregometry	Platelet Rich Plasma is stirred in a cuvette at 37 °C. When an agonist is added, the platelets aggregate increasing light transmission which is detected by a photocell.	>5 mL
PFA-100	Blood is aspirated at high shear rates through cartridges containing an aperture within a membrane coated with either collagen and epinephrine or collagen and ADP. Upon contact, platelets activate, adhere and aggregate occluding the aperture.	800 µL	In vitro thrombus formation under flow	Blood containing fluorescently stained platelets, is pumped at venous shear rates though an aperture coated with collagen. Platelet thrombi formation can be imaged using a fluorescent microscope.	300 µL
			Adhesion	Platelets are added to glass coated with fibrinogen and fixed. Following permeabilization platelets can be imaged using various antibodies and microscopy-based methods.	>5 mL
			Flow cytometry	Fluorescently conjugated monoclonal antibodies are added to blood to label platelets. Platelet agonists are added causing activation. Samples are then passed through a flow chamber equipped with a focused laser beam that activates the fluorophore.	120 µL
			Clot retraction	Thrombin is added to diluted platelet rich plasma and the time taken for a clot to form around a sealed glass pipette is measured.	>5 mL

The overall objective of the study presented here was first to measure platelet function by flow cytometry in premature infants specifically to characterize changes in platelet function in infants according to gestational ages and explore any relationships to the clinical status of the neonates. We also seek to address some issues to pave the way for this assay to transition from a highly specialist centralized laboratories to a near-patient testing procedure that may provide additional guidance in the future for platelet transfusion.

## Methods

### Subjects

The activation status of resting platelets immediately after blood sampling and responses to agonists were studied in 28 neonates, sampled between 0 and 72 h of life. The neonates were subdivided into three gestational age groups: <29 weeks (*n* = 6), 29–36 weeks (*n* = 14) and term babies (defined as >36 weeks gestation, *n* = 8). In addition, within these groups, we also compared results for babies who were defined as suspected septic (*n* = 6) versus healthy (*n* = 22). The minimum number of suspected septic subjects required to achieve 90% power within this study was calculated as five participants. Suspected sepsis was defined based on both clinical suspicion and raised C-reactive protein measurements. Patients characteristic and laboratory results are presented in Tables 2 and 3. All blood samples of neonates were taken after the written informed consent of the parents was given. For some assays assessing sample stability, we used blood obtained by venipuncture from healthy adult volunteers who had given informed consent in accordance with the Helsinki Protocol. This study was given a favourable opinion from the East of England Cambridge Central Research Ethics Committee.

**Table 2:** Summary of patient samples used in this study

Study number	Male/Female	Gestation (weeks)	Platelet count (×10^9^/L)	CRP (mg/L)
1	M	27	229	4
2	F	27	128	4
3	F	28	271	4
4	M	28	146	4
5	M	29	227	4
6	F	29	246	4
7	M	30	238	4
8	M	30	249	4
9	F	32	115	4
10	F	32	328	4
11	F	32	345	4
12	F	32	526	5
13	F	33	349	4
14	M	34	229	4
15	M	34	212	4
16	M	34	243	63
17	F	34	263	4
18	F	34	70	4
19	M	34	215	5
20	F	36	284	4
21	F	37	200	37
22	F	38	397	43
23	M	39	120	27
24	M	40	345	4
25	M	40	329	4
26	M	41	242	4
27	M	41	245	4
28	M	41	319	4

**Table 3:** Summary of patient samples used in this study

	Number	Male	Female	Gestation (weeks)	Platelet count (×10^9^/mL)	CRP (mg/L)
29 weeks and under	6	3	3	28 ± 1	208 ± 72	4 ± 0
30 to 36 weeks	14	6	8	33 ± 3	262 ± 228	8 ± 30
37+ weeks	8	6	2	40 ± 2	276 ± 139	15 ± 20
Healthy	22	12	10	33 ± 7	246 ± 140	4 ± 0
Suspected septic	6	3	3	36 ± 4	284 ± 203	30 ± 29

Data are mean ± range

### Baseline investigations

Full blood counts and C-reactive protein measurement were carried out as part of routine laboratory investigations in the clinical laboratories of the Cambridge University NHS Foundation Trust.

### Blood sampling

We decided not to work with cord blood samples, as premature neonates can spend extended periods of time on the intensive care unit and therefore any assay would need to cover sampling throughout that period. Blood was therefore drawn by venipuncture of a peripheral vein or from an arterial line into a tube containing 3.2% sodium citrate or in a subset of patients, through a capillary. A maximum of 300 µL of blood was taken from each patient.

### Analysis of platelet function

To test fibrinogen binding, 5 µL of citrated whole blood was incubated with a range of concentrations of adenosine diphosphate (ADP, 0.001, 0.01, 0.1, 1 and 10 µM) (Sigma, Gillingham UK) or cross-linked collagen-related peptide (CRP-XL 0.001, 0.01, 0.1, 1 and 10 µg/mL) (Richard Farndale, Cambridge UK) and tested for fibrinogen binding using a polyclonal fluorescein isothiocyanate (FITC) conjugated antibody against fibrinogen (Agilent, Craven Arms UK) (4 µg/mL), followed by incubation at room temperature for 20 min.^8,9,19^ Samples were diluted and fixed with the addition of 0.2% formyl saline (450 µL) and analysed using flow cytometry. To assess fibrinogen binding in the resting platelets we used platelets in ethylenediaminetetraacetic acid (EDTA) as a control sample. EDTA dissociates the αIIbβ3 integrin to which fibrinogen binds, and therefore represents a true negative control.^20^ To test P-selectin exposure 5 µL of citrated whole blood was incubated with the same range of concentrations of ADP or CRP-XL and a phycoerythrin (PE) conjugated monoclonal antibody against P-selectin (CD62-P) (IBGRL Research Products, Bristol UK, clone Thromb6) and a PE-conjugated isotype control (IBGRL Research Products, Bristol UK, clone 9E10) were added (0.4 µg/mL) followed by incubation for 20 min.^8,9,21–23^ Samples were then diluted and fixed as for the fibrinogen assays and analysed using flow cytometry.

### Flow cytometry

For each sample, the fluorescence from 5000 platelets, gated by their characteristic forward and side scatter (Supplementary Figure S1A (online)),^24–26^ was measured on a FACSVia flow cytometer (BD Biosciences, Oxford UK). For the analysis of fibrinogen binding, percentage positive platelet values were determined by gating 2% of the negative population using EDTA (10 µM) (Sigma, Gillingham UK) (Supplementary Figure S1B (online)). For P-selectin exposure, the mean fluorescence intensity (MFI) was calculated from the platelet population as defined by the forward and side light scatter (Supplementary Figure S1C (online)). Data were acquired using BD FACSVia research loader software (BD Biosciences, Oxford UK) and analysed using Kaluza version 1.5 (Beckman Coulter, High Wycombe, UK). The stability of performance of the flow cytometer was tested daily using FACSVia Cytometer Setup and Tracking beads (BD Biosciences, Oxford UK).

### Statistics

The primary end points of the study were to record platelet responses to ADP and CRP-XL agonists in the form of fibrinogen binding and P-selectin exposure for the different gestational ages and suspicion of sepsis. Results are presented either in scatter graphs or box plots indicating mean values of all subjects in the defined groups ± standard error of the mean (SEM). In order to capture the dynamics of dose response curve to each agonist in one value, we calculated the EC_50_. The multiple readouts across the range of agonist concentrations were baselined (such as that resting value represent 0% response), and the data normalized (such that 100% response is the maximal response to agonist). From this, a non-linear regression curve was generated and the EC_50_ was deduced from these curves as the concentration of agonists necessary to obtain either 50% of platelets bound to fibrinogen or a 50% increase in the MFI for P-selectin exposure between the resting sample and the sample with maximal response (Supplementary Figure S2 (online)). The Fibrinogen Binding Score was generated by adding the ADPmax and CRPmax % Fibrinogen-bound platelets. The Degranulation Score was generated by adding ADPmax and CRPmax P-selectin exposure MFI. Statistical analysis was performed in GraphPad Prism (GraphPad Software, La Jolla California) achieved by unpaired *t* testing or two-way ANOVA. *P* < 0.05 was regarded as significant.

## Results

### Validation of a near-patient standardized assay

#### Sample type

**Fig. 1:**
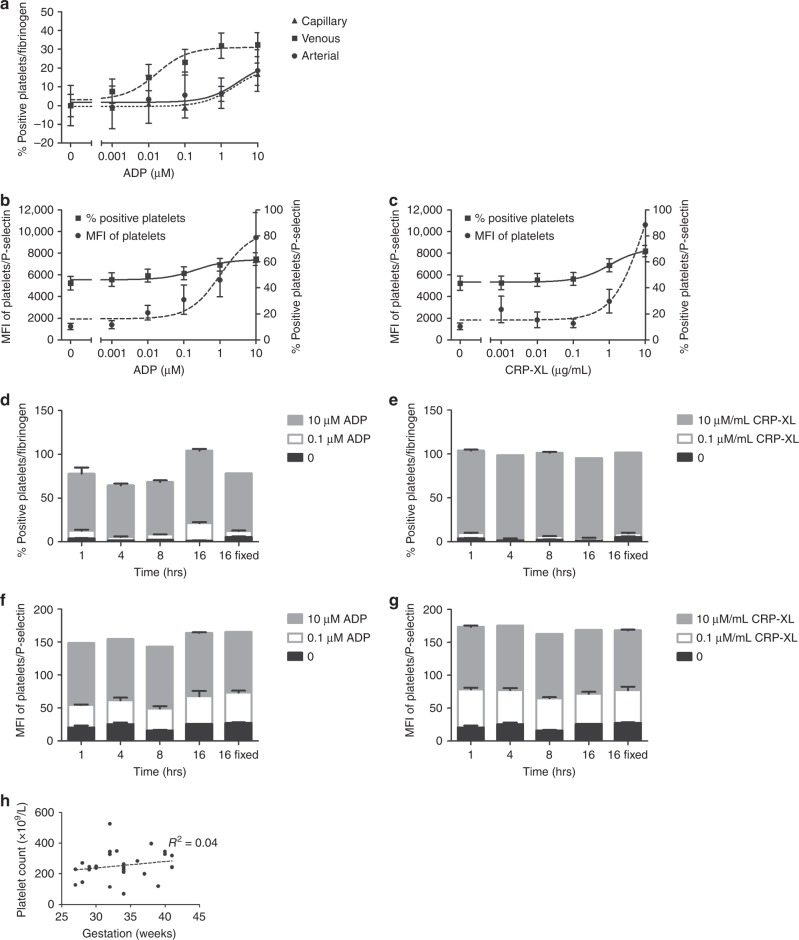
The generation of agonist dose response curves from neonatal platelets. **a** Blood from neonatal donors from an arterial line, capillary or vein was incubated with ADP and platelet fibrinogen binding was measured (mean, ± SEM). **b**, **c** Platelets from 28 neonates were activated with ADP (**b**) or CRP-XL (**c**) and P-selectin exposure was measured as percentage of positive platelets or platelet mean fluorescence intensity (MFI) (mean, ± SEM). **d**–**g** Platelets from three healthy adult donors were activated with ADP (**d**, **f**) or CRP-XL (**e**, **g**) and percentage of fibrinogen binding (**d**, **e**) or MFI of exposed P-selectin (**f**, **g**) was measured (mean, ±SEM). Two lag delay periods were artificially introduced: (1) time between sample fixation and running the sample on the flow cytometer (1, 4, 8 and 16 h), and(2) the time between venipuncture and platelet stimulation extended to 16 h, after which they were immediately fixed and read on the flow cytometer. **h** Platelet count was plotted against the gestation of 28 neonates and the linear regression co-efficient (*R*^2^) was calculated

Capillary and arterial samples were considerably less responsive to agonists than venous samples (Fig. 1a), though this muted response was overcome at the highest concentration of agonist. Consequently, only venous samples were used for this study.

#### Measurements of activation

Adult blood samples for measurement of platelet function by flow cytometry are traditionally taken under optimal conditions, with individuals often resting prior to venipuncture and the use of wide bore needles, without a tourniquet, to avoid platelet activation resulting from the sampling itself. As these conditions are not appropriate for premature neonates we looked at baseline activation of the neonatal platelets in our study. We found that baseline fibrinogen binding was below 10% in all samples bar the neonates in the lowest age group (see below) and that there was an increase in the percentage of platelets binding fibrinogen when they were activated with increasing concentration of ADP or CRP-XL that would allow us to analyse the dynamics of the platelet activation. By contrast. we found that the percentage of platelets showing P-selectin surface expression was already raised to around 50% in all resting samples when compared to an isotype control, meaning that only a very shallow curve was produced on increasing agonist concentrations, making analysis impossible. Instead, we show that using the MFI of the whole platelet population with the P-selectin antibody restores the dose response curve to ADP and CRP-XL allowing for meaningful analysis of the data (Fig. 1b, c). Conversely using MFI for fibrinogen binding produced muted dose response curves and so throughout this study percentage positive platelets was used for fibrinogen binding and MFI for P-selectin exposure.

#### Stability of the samples

When samples are taken in a busy clinical setting there may be a delay between sampling and carrying out the assay. Similarly, there may be a delay in between the moment the samples are fixed at the end of the activation reaction and when they are actually analysed on the flow cytometer. To ascertain whether any of these lag periods influenced the results obtained with the flow cytometry assay, we used blood obtained from three healthy adult volunteers and artificially set time delays between venipuncture, platelet stimulation and running the fixed samples on the flow cytometer. We show that baseline activation and response were not influenced by either of these two lag periods (Fig. 1d–g, *P* > 0.05 by ANOVA).

#### Platelet count and function

Premature neonates are often found to be thrombocytopenic, especially with added co-morbidity such as sepsis. Using platelet flow cytometry to analyse platelet function should therefore not be influenced by platelet count. In this cohort of patients, the mean platelet count was 254 × 10^9^/L with a range from 70 to 526 × 10^9^/L (Table 3). There was no correlation between platelet count and gestational age (Fig. 1e) or EC_50_ results (Table 4).

**Table 4:** *R*^2^ values to assess the correlation between EC_50_ values of responses to ADP and CRP-XL versus the platelet count of all 28 neonates

	Agonist and measurement	*R* ^2^
% Positive platelets	ADP Fibrinogen	−0.17273459
	CRP-XL Fibrinogen	−0.010157365
MFI of platelets	ADP P-selectin	−0.016622008
	CRP-XL P-selectin	−0.120926249

Validation of the methodology used for the measurement of platelet function in neonates, measuring the stability of the samples and finding no correlation between platelet function and count, allowed a more detailed investigation of neonatal platelet function.

### Impact of gestational age on neonatal platelet function

#### Fibrinogen binding responses from neonatal platelets from different gestational age groups

**Fig. 2:**
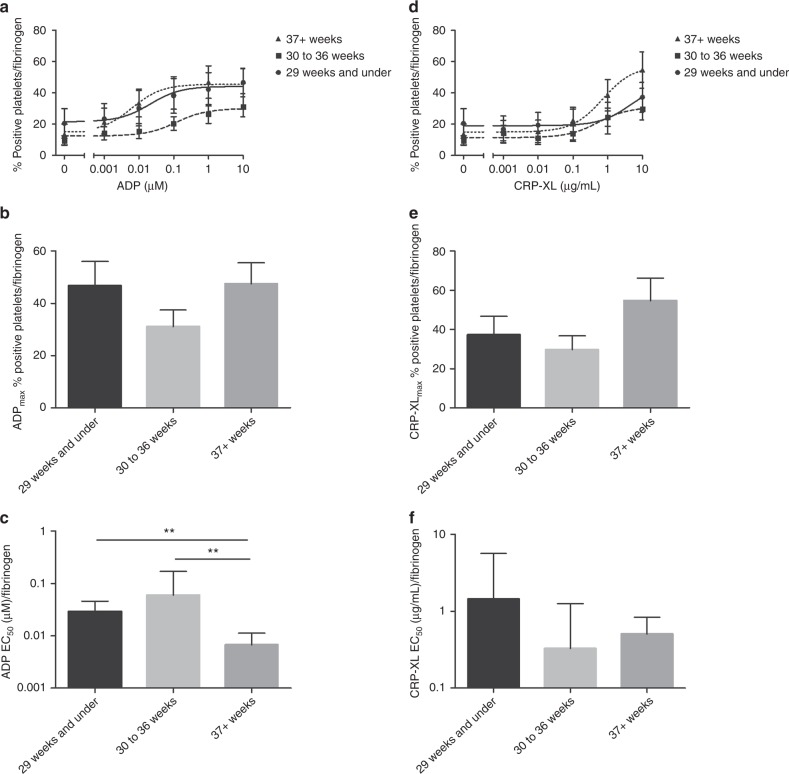
Fibrinogen binding to resting and activated platelets from neonates born at different gestational ages. Platelets from 28 neonates were activated with ADP **a**–**c** or CRP-XL **d**–**f** and fibrinogen binding was measured by flow cytometry. Neonates were divided according to their gestational age and mean dose response curves (mean ± SEM) are shown using either ADP (**a**) or CRP-XL (**d**) as an agonist. Platelet response to ADP_max_ (10 µM) (**b**) or CRP-XL_max_ (10 µg/mL) (**e**) are shown (mean ± SEM). The EC_50_ values were calculated from baseline removed, normalize, non-linear regression curves (**c**, **f**, mean ± 95% confidence interval)

The percentage of platelets binding fibrinogen was recorded in the resting samples and in response to a range of concentrations of ADP (0.001 to 10 μM) and CRP-XL (0.001 to 10 μg/mL). Platelets of neonates born at 29 weeks or shorter gestation had a higher resting level of fibrinogen binding (20.5% ± 9) when compared to neonates born between 30 and 36 weeks (9.5% ± 3) and term neonates (13.5% ± 6). The dose response curves according to gestational age groups in response to ADP are presented in Fig. 2a. At the highest concentration of ADP used, fibrinogen binding was at a similar percentage for the term babies and neonates born before 29 weeks gestation but slightly lower in the middle group (46.7% ± 9 in the <29 weeks gestation vs 31.1% ± 6 and 47.5% ± 9 for the 30–36-week gestation and term neonates, respectively, Fig. 2b). The EC_50_ ADP/Fibrinogen value was significantly higher in the less than 29 weeks group (0.03 µM ± 0.02) and the 30-36 weeks group (0.06 µM ± 0.1) compared to the term neonates (0.007 µM ± 0.005, *P* = 0.0004 and *P* = 0.0009 respectively) meaning that the neonates in the lower gestational age groupings responded less well than term neonates (Fig. 2c).

The dose response curves to CRP-XL according to gestational age groups is shown in Fig. 2 and whilst the same trends were observed as for ADP, this did not reach significance.

#### P-selectin exposure from neonatal platelets from different gestational age groups

**Fig. 3:**
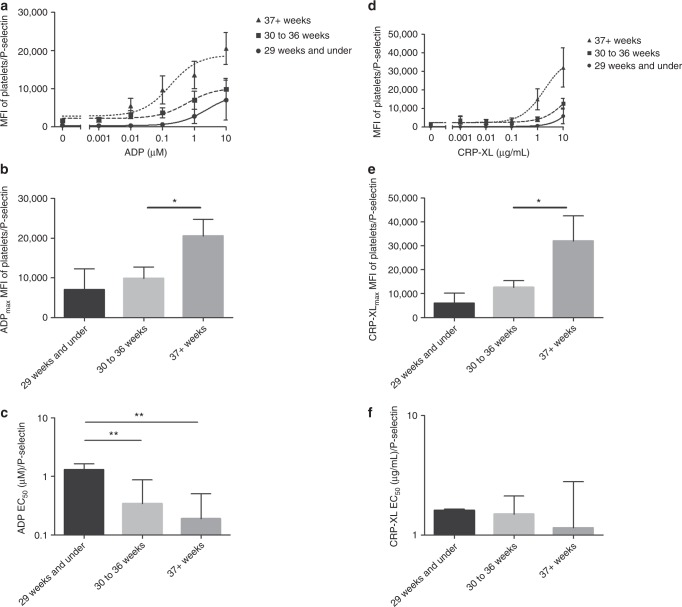
P-selectin exposure on resting and activated platelets from neonates according to gestational ages. Platelets from 28 neonates were activated with ADP (**a**–**c**) or CRP-XL (**d**–**f**) and P-selectin exposure was measured by flow cytometry. Neonates were divided according to their gestational age and mean dose response curves (mean ± SEM) are shown using either ADP (**a**) or CRP-XL (**d**) as an agonist. Platelet response to ADP_max_ (10 µM) (**b**) or CRP-XL_max_ (10 µg/mL) (**e**) are shown (mean ± SEM). The EC_50_ values were calculated from baseline removed, normalize, non-linear regression curves (**c**, **f**, mean ± 95% confidence interval)

The total level of P-selectin exposure (measured by the MFI of the total platelet population) was similar in the three different gestational groups for the resting platelets. However, the rate of increase of the MFI with increasing concentrations of ADP correlated with gestational age (Fig. 3a). This is best reflected in the differences in the MFI at maximum concentration of ADP, which was higher in the term babies compared to premature babies. This difference reached significance for the 30–36 weeks group when compared to the term neonates (9859 ± 2884 vs 20,538 ± 4170, *P* = 0.04) and near-significance (*P* = 0.06) for the <29 weeks gestation group (Fig. 3b). The EC_50_ ADP/P-selectin was significantly higher in the <29-week group (0.13 µM ± 0.35) compared to both the 30–36 week (0.34 µM ± 0.53) and term neonates (0.19 µM ± 0.32) (*P* = 0.0002 and *P* = 0.001, respectively, Fig. 3c), confirming the decreased response to ADP in the very premature babies. P-selectin exposure in response to CRP-XL showed a similar trend where the MFI at maximum concentration of CRP-XL was higher in the term neonates compared to the premature babies, which again reached significance for the 30–36-week group (12,549 ± 2877 vs 32,030 ± 10,535, *P* = 0.03) and near significance in the <29-week group (*P* = 0.07, Fig. 3d, e). No significant differences in the EC_50_ of CRP-XL/P-selectin were observed (Fig. 3f).

Overall, P-selectin exposure proved a more sensitive measurement of platelet function as the EC_50_ ADP, and the highest concentration of both ADP and CRP used were significantly different.

### Impact of sepsis on neonatal platelet function

A subset of six neonates used within this study were identified as suspected septic based on clinical suspicion and a plasma C-reactive protein levels above 4 mg/L (range from 5 to 63 mg/L with a mean of 30 mg/L).

#### Platelets from suspected septic babies show decreased fibrinogen binding

**Fig. 4:**
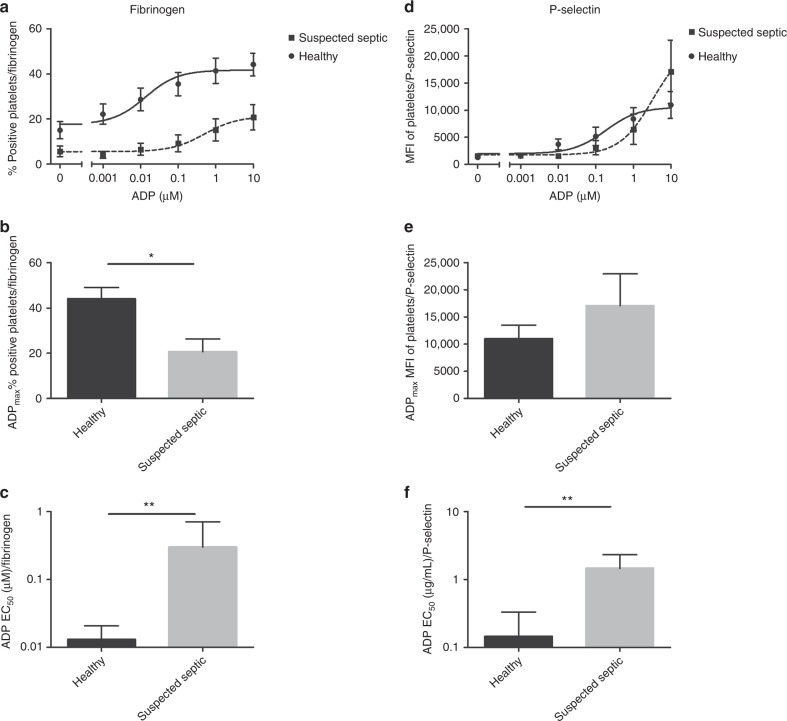
Fibrinogen binding and P-selectin exposure on resting and activated platelets from suspected septic and healthy neonates. Platelets from 28 neonates were activated with ADP and fibrinogen binding (**a**–**c**) or P-selectin exposure (**d**–**f**) was measured by flow cytometry. Neonates were divided into suspected septic or healthy group and mean dose response curves (mean ± SEM) are shown using ADP as an agonist (**a**, **d**). Platelet response to ADP_max_ (10 µM) are shown (mean ± SEM) (**b**, **e**). The EC_50_ values were calculated from baseline removed, normalize, non-linear regression curves (**c**, **f**, mean ± 95% confidence interval)

Suspected septic neonates showed a lower percentage of fibrinogen bound platelets both at rest (5.4% ± 2 vs 15% ± 4 in the suspected septic and healthy babies respectively) and at the maximal concentration of ADP (20.7% ± 4 vs 44.1% ± 6, *P* = 0.03) when compared to the healthy neonates (Fig. 4a, b). This reduced response to ADP in the suspected septic neonates was reflected in the mean EC_50_ ADP/Fibrinogen values which were increased in the suspected septic neonates (0.3 µM ± 0.41 vs 0.01 µM ± 0.08, *P* = 0.0001) (Fig. 4c). Similar trends were observed with platelet responses when fibrinogen binding was measured with the maximum concentration of CRP-XL and EC_50_ CRP-XL; however, this did not reach the threshold of significance (Supplementary Materials and Methods and Figure S3A, B and C (online)).

#### Platelets from suspected septic babies require higher concentrations of agonist to expose P-selectin

P-selectin exposure at rest was not different between suspected septic and healthy neonates (Fig. 4d). Although the suspected septic neonates showed a higher response at the maximum ADP concentration this was not statistically significant (Fig. 4e). However, the EC_50_ ADP/P-selectin was significantly lower in the healthy group compared to the suspected septic neonates (0.1 µM ± 0.19 vs 1.46 µM ± 0.87, *P* = 0.0001) (Fig. 4f) indicating that platelets from babies with a potential infection are less responsive than those from healthy babies. No significant differences were observed with P-selectin expression in response to the maximum concentration of CRP-XL or EC_50_ CRP-XL between suspected septic and healthy babies (Supplementary Materials and Methods and Figure S3D, E and F (online)).

The use of ADP as an agonist to detect differences between healthy and suspected septic neonates was superior to CRP-XL, as EC_50_ ADP/fibrinogen binding, and the highest concentration of ADP/P-selectin and EC_50_ ADP/P-selectin were significantly different.

### Combining the ADP and CRP-XL responses at maximum concentration identifies clearly separate clinical groups

**Fig. 5:**
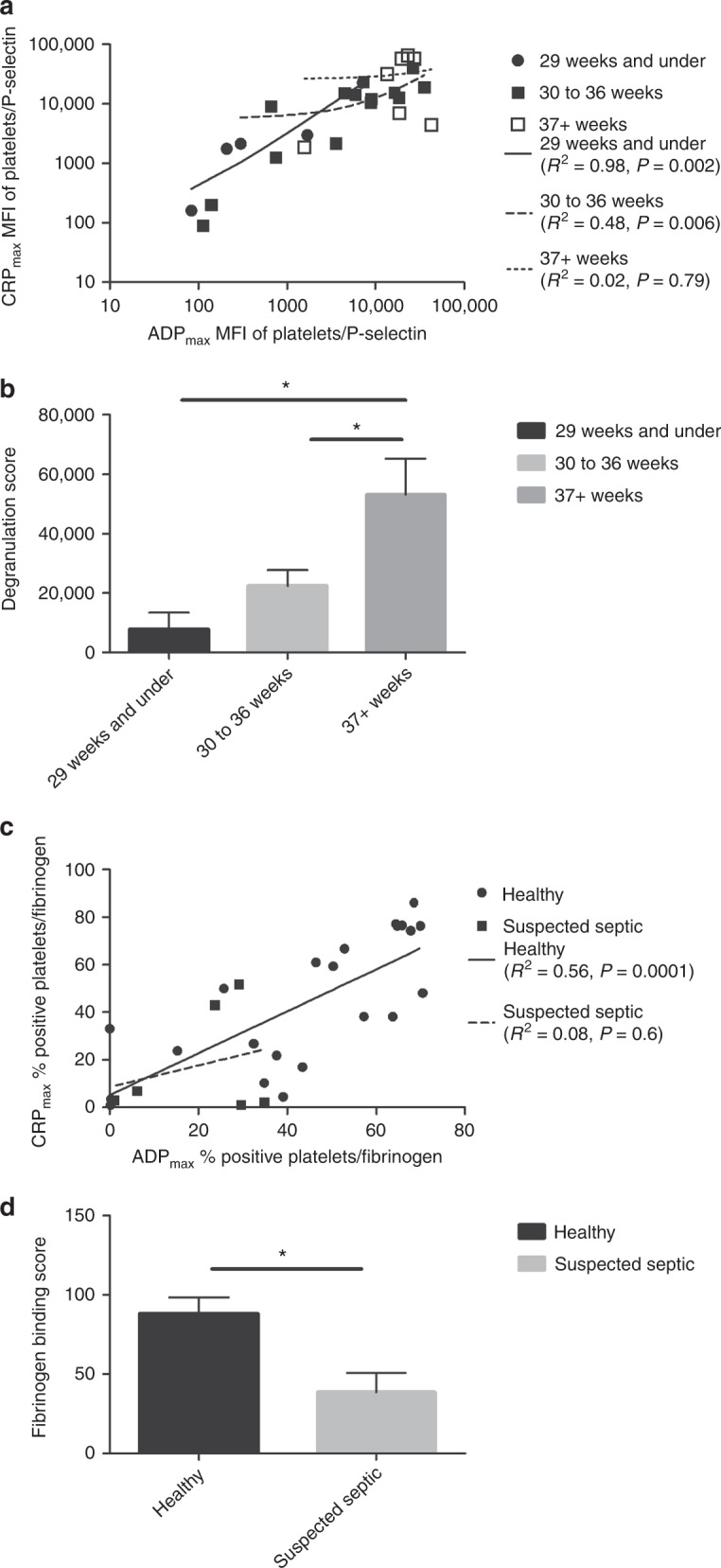
Combining ADP and CRP-XL-mediated activation of neonatal platelets identifies clear clinical groups. MFI of platelets with exposed P-selectin at ADP_max_ and CRP-XL_max_ (**a**, **b**). **b**)ADP_max_ was added to CRP-XL_max_ to generate a Degranulation Score. Results are presented as mean values ± SEM. Percentage positive fibrinogen bound platelets at ADP_max_ and CRP-XL_max_ (**c**, **d**). **d** ADP_max_ was added to CRP-XL_max_ to generate a Fibrinogen Binding score. Results are presented as mean values ± SEM

To be clinically relevant, the flow cytometry data should be able to distinguish between groups of neonates with different clinical characteristics, in this case gestational age or sepsis. The data presented above suggest that the read-outs for maximum concentrations of agonists may be used for this purpose. Figure 5a shows for each individual neonate the distribution of the MFIs for P-selectin exposure at the maximum dose of ADP (10 µM) and the maximum dose of CRP-XL (10 µg/mL). The correlation coefficient (*R*^2^) between the two measurements did not significantly correlate in the term neonates (*R*^2^ = 0.02), whilst in the 30–36-week group they weakly but significantly correlated (*R*^2^ = 0.48, *P* = 0.006) and in the 29 weeks and under group these two read-outs had a strong positive correlation (*R*^2^ = 0.98, *P* = 0.002) (Fig. 5a). This correlation in the younger neonates was reflected when both read-outs were combined into a Degranulation Score (in effect the addition of ADPmax and CRPmax P-selectin exposure MFIs) which was significantly lower in both the very premature neonates and neonates born between 30 and 36 weeks than the term neonates (7837 ± 5548 and 22,408 ± 5502 vs 48,565 ± 11433, *P* = 0.01 and *P* = 0.03, respectively, Fig. 5b).

We went on to see whether a similar observation could also be made for the suspected septic vs healthy babies, this time using the fibrinogen binding response at ADP_max_ and CRP_max_. Healthy neonates showed a moderate correlation between the two measurements (*R*^2^ = 0.56, *P* = 0.0001), whilst the suspected septic neonates had no correlation (*R*^2^ = 0.08) (Fig. 5c). When both measurements were combined into a Fibrinogen Binding Score (again adding the ADPmax and CRPmax % Fibrinogen-bound platelets), we found it was significantly lower in suspected septic neonates (39 ± 12.2 vs 88 ± 10.3, *P* = 0.03) when compared to the healthy neonates (Fig. 5d).

Incidentally, the same analyses were performed using fibrinogen binding vs gestational age and P-selectin exposure vs suspected sepsis but no significant differences were observed (data not shown). Combining the functional analysis further supports the observation that platelet function varies according to both gestational age and clinical status.

## Discussion

There have been so far very few attempts to correlate platelet function in neonates with clinical conditions that are known to increase the risk of bleeding such as sepsis, in particular necrotizing enterocolitis. Even further, the idea that measuring platelet function by the bedside may add an extra level of guidance to the clinicians when the decision is taken to transfuse platelets to the neonates has not been considered, as flow cytometry has traditionally been an assay that has required highly trained staff, specialist equipment in centralized laboratories, and bespoke analyses making it unsuitable for such an application. Our findings support a potential novel means to routinely assess platelet function analysis by flow cytometry in preterm neonates in intensive care units, who are at very high risk of bleeding and where the usual laboratory tests of platelet count have limitations to predict bleeding risk. We showed that the latest generation of small footprint clinical flow cytometers, no longer requiring large dedicated laboratory bench space and often used for immunological investigations such as quantification of CD4+ cells for HIV clinics, can be used for platelet assays. Furthermore, inherent software developments and automation have reduced the need for highly skilled dedicated operators. The platelet assays were robust enough to give consistent results across a range of lag times between sampling and assaying, inevitable in clinical situations. These assays have previously been used in the study of adult platelet function, however these were conducted in relatively controlled settings and environments which would be unachievable for the study of neonatal platelets. This prompted a re-evaluation and adoption of novel analysis methods which were able to identify significant differences between the different gestation age or clinical groups studied.

In addition, during the performance of this study we continually reviewed ways of refining analysing platelet function in neonates to produce a methodology better suited to near-patient testing. Manual tube-based tests are a source of variability, especially when considering multiple operators. However other groups have used pre-aliquoted reagents freeze dried in microplate format for adult platelet function studies. These have the potential to further improve and standardize testing by using the flow cytometers’ microplate sampling capacity to both reduce operator related variability and increase the range of agonists tested. Towards the end of the study pre-loaded microplates became available to us for testing and were utilized for samples from four neonates (Supplementary Materials and Methods and Figure S4 (online)). Whilst the results demonstrated anticipated dose response curves these plates were designed for use with adult platelets and so a higher dose of agonist would be required, as indicated in this study using a tube-based assay, to study neonatal platelet function. By increasing the range of concentrations, however, along with the addition of other platelet agonists (e.g. thrombin receptor activating peptide and epinephrine) and considering their potential for reducing variation and increasing automation, their inclusion into future test systems would be beneficial. Robotics have also progressed so much that it is entirely conceivable that small volume of whole blood samples from neonates could be added to preloaded plates without the need for a skilled operator. The ultimate goal of “sample in/result out” is entirely conceivable with existing technologies.

We focused on platelet response to ADP and CRP-XL looking at fibrinogen binding and P-selectin exposure because these agonists and markers of platelet activations reflect different platelet activation pathways and functional responses and have been previously shown to sensitively and reproducibly detect small platelet function variations in a large study of healthy adult volunteers.^27,28^ We were particularly looking at detecting potential differences between neonates born at different gestational age and in neonates who were diagnosed as suspected septic based on clinical suspicion and raised CRP plasma levels. We found that platelets from premature babies exposed less P-selectin on their surface. In addition, the analysis of the dynamic response across the range of agonists concentrations (reflected in the EC_50_) suggested that platelets from premature babies responded less readily to ADP than those from term neonates. When looking at the suspected septic babies, we found a greatly reduced response to ADP together with a reduced activation of the fibrinogen receptor across the range of both ADP and CRP-XL. A presumptive diagnosis of early-onset neonatal sepsis (EONS) in the immediate period following birth without confirmatory positive blood cultures may cover a range of pathogenic conditions. This includes infections acquired from the chorioamniotic fluid with fastidious organisms that do not grown in culture or a “general inflammatory state” created by cytokines and/or endotoxins acquired from the amniotic fluid.^29^ Regardless of the etiology our data suggest an effect on platelet function, which correlates with previous observations that sepsis is a risk factor for IVH in newborns.

It is difficult to compare data across studies as analyses of raw flow cytometric data can vary enormously from one study to the other and interpretation are dependent on the use of appropriate controls. For example, in this study we used EDTA-treated platelets to demonstrate a clear increase in fibrinogen binding in the resting platelets. In addition, sampling can play a major role in platelet activation assays. We demonstrate that, in our hands, heel prick samples are considerably less responsive than samples obtained by venipuncture and from indwelling lines, therefore heel prick samples may lead to an apparent “blunted” platelet response and mask differences between individuals.

Future research requires a rigorous assessment of flow cytometry functional data, alongside clinical data, including bleeding complications and use of platelet transfusions. Our results have demonstrated the feasibility of using flow cytometry in a near-patient setting on a ward, rather than in a large centralized laboratory facility. The use of standardized pre-loaded reagent plates would support comparison of data between patients and between each neonatal unit, allowing for multicenter studies. In addition, we show that combination of data to generate a single output reading (such as the Degranulation Score and Fibrinogen Binding Score) clearly identifies different subtypes of platelet function profile linked to clinical data such as gestational age and sepsis. It is therefore entirely possible that such single output reading may be correlated to bleeding risk and therefore guide platelet transfusion. To this end, a larger likely multicentre study design, would be needed, where bleeding score would be recorded alongside flow cytometry data. This approach would simplify data analysis for the clinician as it would not require dose response curve interpretation and would be more amenable to setting up trigger criteria for transfusion.

## Supplementary information

Supplementary Figure S1

Supplementary Figure S2

Supplementary Figure S3

Supplementary Figure S4
