# Supplementary figures and images for: Flow cytometry for near-patient testing in premature neonates reveals variation in platelet function: a novel approach to guide platelet transfusion
Source: Pediatr Res. 2019 Jan 29;85(6):874–84. doi: 10.1038/s41390-019-0316-9 (PMC6760564; doi:10.1038/s41390-019-0316-9)

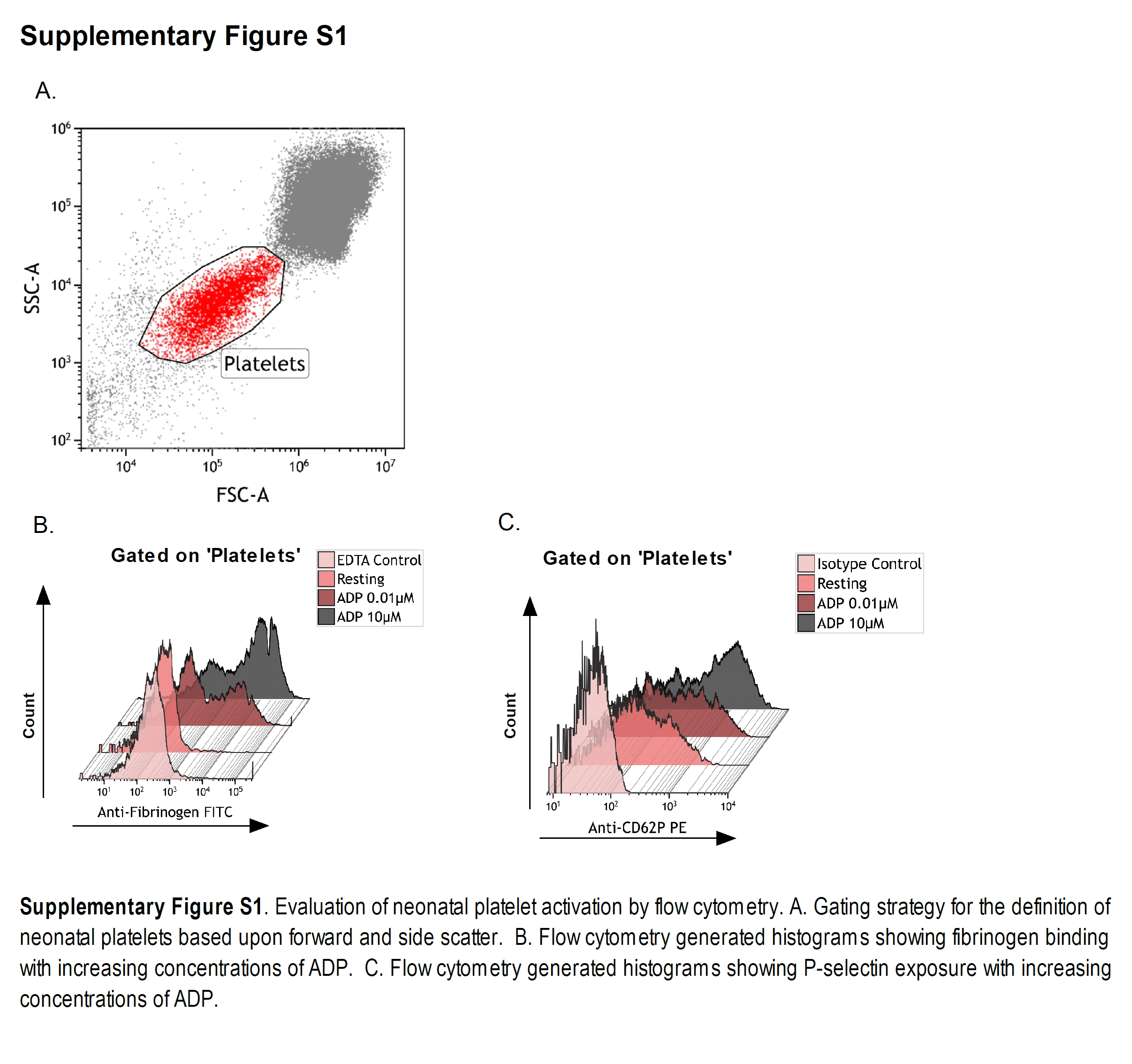

Supplement: Supplementary file 1 — Supplementary Figure S1 [file 41390_2019_316_MOESM1_ESM.tif]

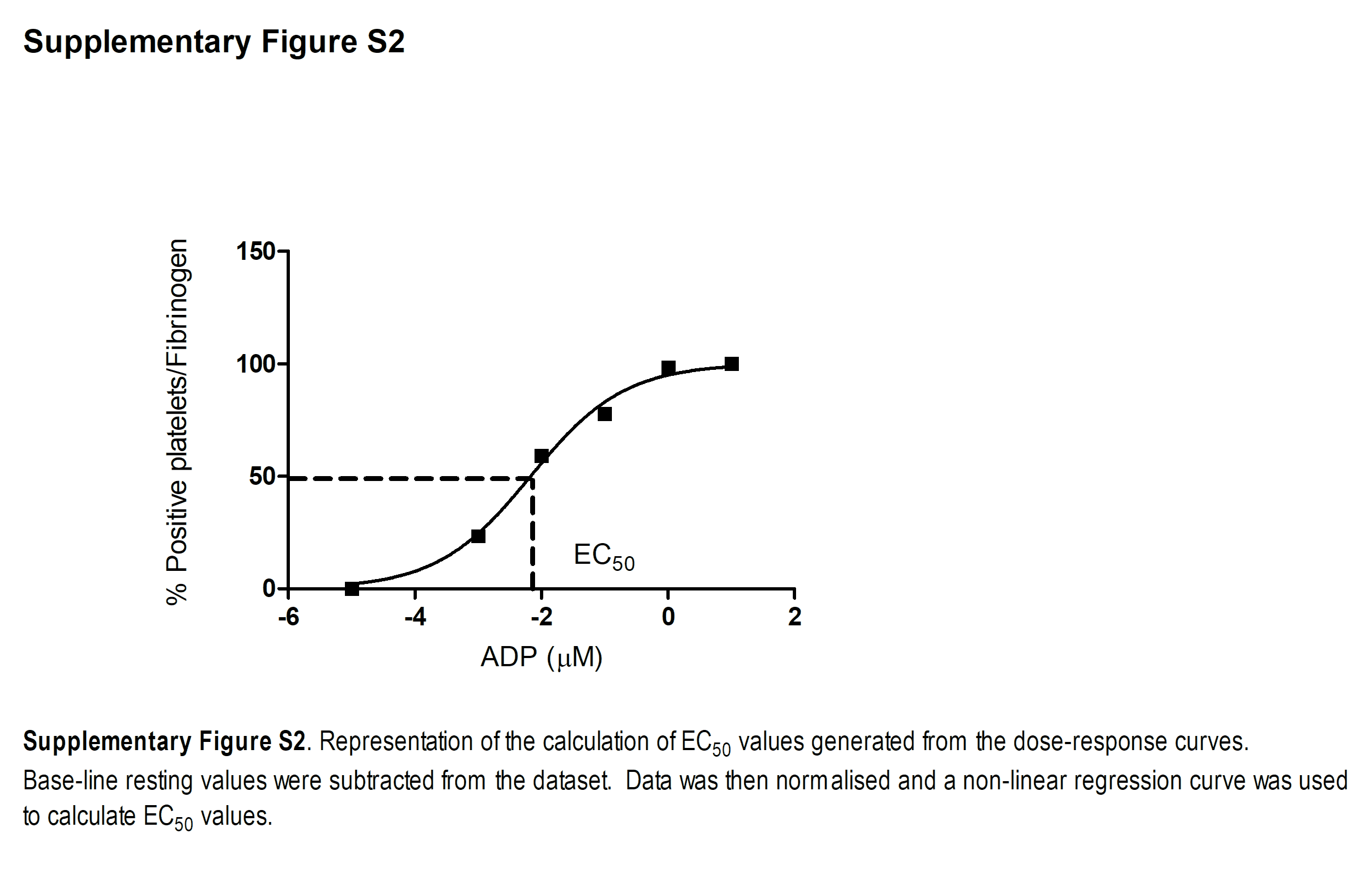

Supplement: Supplementary file 2 — Supplementary Figure S2 [file 41390_2019_316_MOESM2_ESM.tif]

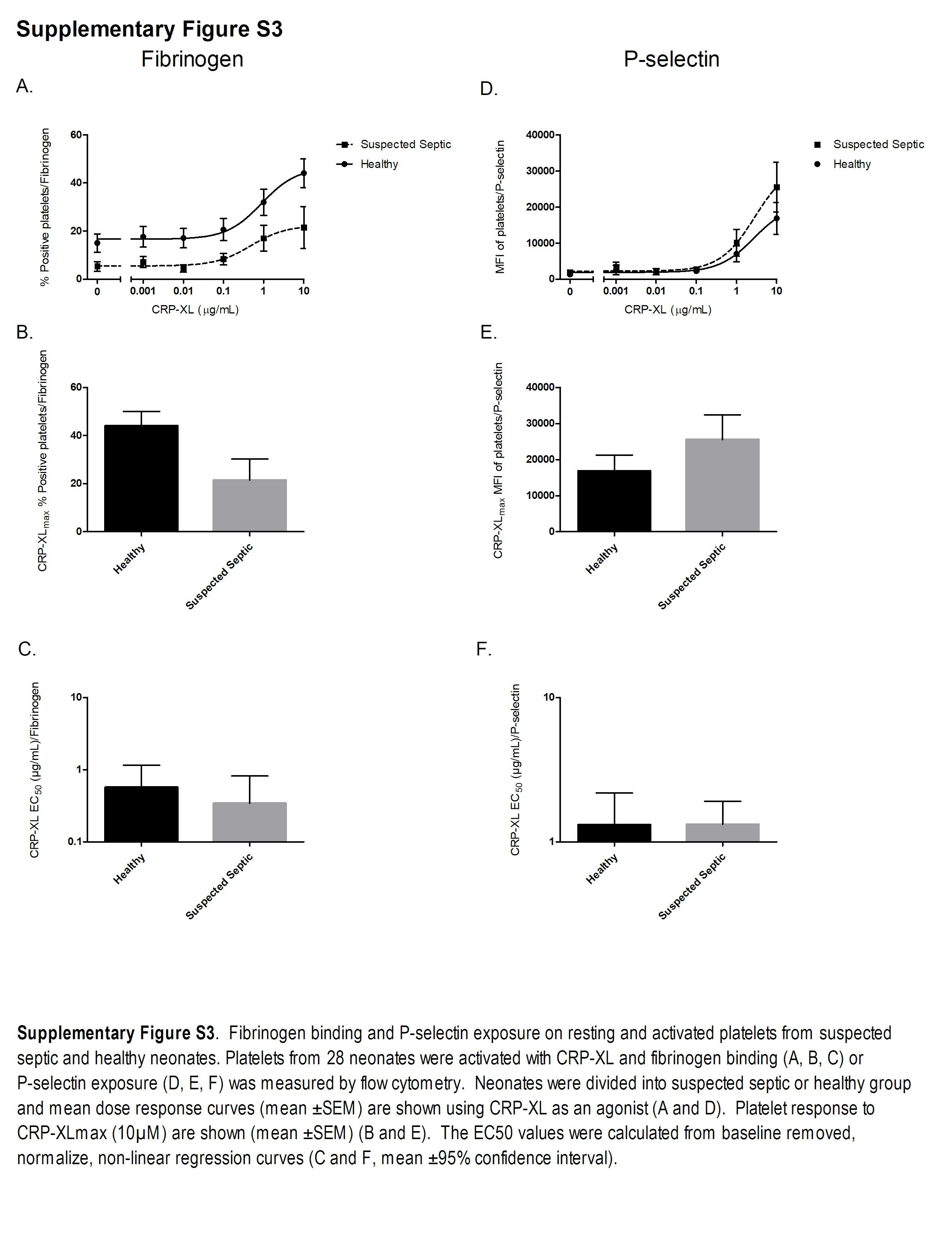

Supplement: Supplementary file 3 — Supplementary Figure S3 [file 41390_2019_316_MOESM3_ESM.tif]

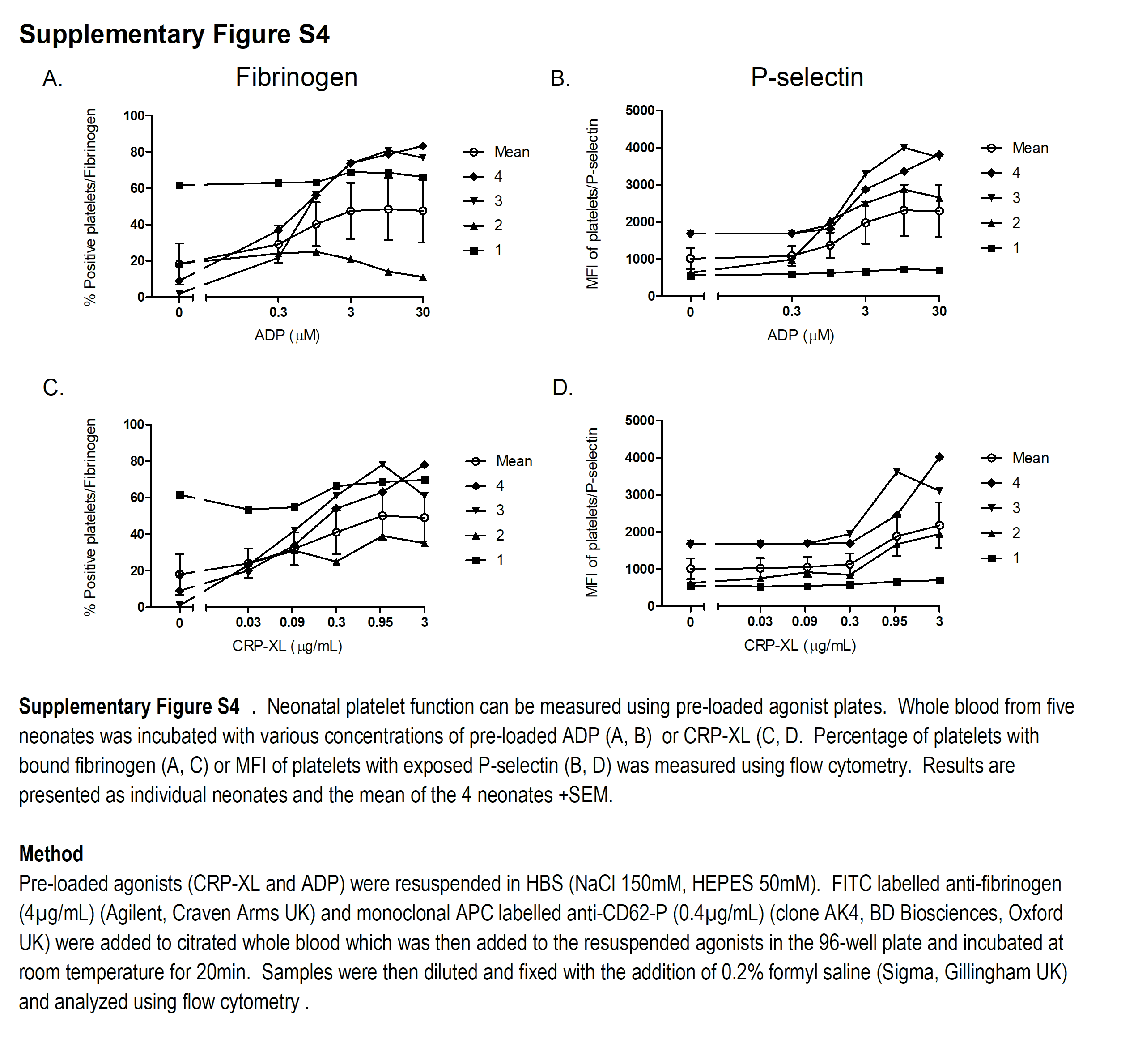

Supplement: Supplementary file 4 — Supplementary Figure S4 [file 41390_2019_316_MOESM4_ESM.tif]
